# Altering the interfacial rheology of *Pseudomonas aeruginosa* and *Staphylococcus aureus* with N-acetyl cysteine and cysteamine

**DOI:** 10.3389/fcimb.2023.1338477

**Published:** 2024-01-16

**Authors:** Sricharani Rao Balmuri, Sena Noaman, Huda Usman, Tagbo H. R. Niepa

**Affiliations:** ^1^ Department of Chemical and Petroleum Engineering, Pittsburgh, PA, United States; ^2^ Center for Medicine and the Microbiome, Pittsburgh, PA, United States; ^3^ The McGowan Institute for Regenerative Medicine, University of Pittsburgh, Pittsburgh, PA, United States; ^4^ Department of Chemical Engineering, Pittsburgh, PA, United States; ^5^ Department of Biomedical Engineering, Carnegie Mellon University, Pittsburgh, PA, United States

**Keywords:** *Pseudomonas aeruginosa*, *Staphylococcus aureus*, fluid interfaces, thin-film, interfacial tension (IFT), viscoelastic materials, N-acetyl cysteine, cysteamine

## Abstract

**Introduction:**

Chronic lung infection due to bacterial biofilms is one of the leading causes of mortality in cystic fibrosis (CF) patients. Among many species colonizing the lung airways, *Pseudomonas aeruginosa* and *Staphylococcus aureus* are two virulent pathogens involved in mechanically robust biofilms that are difficult to eradicate using airway clearance techniques like lung lavage. To remove such biological materials, glycoside hydrolase-based compounds are commonly employed for targeting and breaking down the biofilm matrix, and subsequently increasing cell susceptibility to antibiotics.

**Materials and methods:**

In this study, we evaluate the effects of N-acetyl cysteine (NAC) and Cysteamine (CYST) in disrupting interfacial bacterial films, targeting different components of the extracellular polymeric substances (EPS). We characterize the mechanics and structural integrity of the interfacial bacterial films using pendant drop elastometry and scanning electron microscopy.

**Results and discussion:**

Our results show that the film architectures are compromised by treatment with disrupting agents for 6 h, which reduces film elasticity significantly. These effects are profound in the wild type and mucoid *P. aeruginosa*, compared to *S. aureus*. We further assess the effects of competition and cooperation between *S. aureus* and *P. aeruginosa* on the mechanics of composite interfacial films. Films of *S. aureus* and wild-type *P. aeruginosa* cocultures lose mechanical strength while those of *S. aureus* and mucoid *P. aeruginosa* exhibit improved storage modulus. Treatment with NAC and CYST reduces the elastic property of both composite films, owing to the drugs’ ability to disintegrate their EPS matrix. Overall, our results provide new insights into methods for assessing the efficacy of mucolytic agents against interfacial biofilms relevant to cystic fibrosis infection.

## 1 Introduction

Cystic fibrosis is a genetic disorder caused by mutations in the cystic fibrosis transmembrane regulator (CFTR) gene. The CFTR gene encodes for a protein that functions as a voltage-gated channel for the transport of chloride ion across the cells. Mutation in the CFTR gene leads to the transcription of a dysfunctional protein, which is incapable of maintaining the cell’s homeostasis due to impaired transport of chloride ions (Marks, 1990; Ahlgren et al., 2015; Bernardy et al., 2020; Briaud et al., 2020; Fischer et al., 2021). In the lungs, this leads to mucus buildup on the epithelial cells, impairing mucociliary clearance (Robinson and Bye, 2002; Mall and Hartl, 2014). The ensuing interfacial environment favors the growth and proliferation of various microbes like *P. aeruginosa, S. aureus, Haemophilus influenzae, Burkholderia cepacia*, and *Strenotrophomonas maltophilia* (Harrison, 2007). Consequently, chronic infections build up in the lungs of CF patients, as the pathogens undergo pathoadaptation and develop multidrug resistance. For instance, *P. aeruginosa* alters its cellular and genetic functions to mitigate antibiotic targets and form biofilms, which act as a biological shield against antimicrobials (López-Causapé et al., 2015; Høiby et al., 2017).

Biofilm formation in cystic fibrosis (CF) patients, primarily driven by *P. aeruginosa* and *S. aureus*, significantly impacts clinical outcomes (Marks, 1990; de Boer et al., 2011; Ahlgren et al., 2015; Bernardy et al., 2020; Briaud et al., 2020; Fischer et al., 2021). These pathogens, prevalent in CF patients’ lungs, invade the airways, and their presence in the sputum correlates with infection severity (2021; Hubert et al., 2013; Beaudoin et al., 2017; Cystic Fibrosis Foundation Patient Registry, 2022). In the early stage of the infection, both species exhibit antagonist behavior, with *P. aeruginosa* secreting rhamnolipids, toxins, and secondary metabolites such as 2-heptyl-4-hydroxyquinoline-N-oxide (HQNO) to inhibit, lyse, and outcompete *S. aureus* cells (Biswas et al., 2009; Filkins et al., 2015; Beaudoin et al., 2017; Bernardy et al., 2020; Camus et al., 2021). However, in the later stage of CF infection, both strains coexist due to adaptations that minimize this antagonistic behavior (Limoli et al., 2017; Price et al., 2020). *P. aeruginosa* undergo a mucoid switch, leading to the overproduction of alginate polysaccharides whilst reducing its anti-staphylococcal activity. On the other hand, *S. aureus* mutates to a small-colony variant (SCV) phenotype, where its fermentative adaptation enables high resistance to antibiotics and survival against *P. aeruginosa* attacks (Biswas et al., 2009). Clearly, these phenotypic shifts enhance biofilm formation with both species achieving a balance. Therefore, elucidating the dynamics of *P. aeruginosa* and *S. aureus* and their contribution to biofilms at various stages of infection is important to develop antibiofilm strategies against multispecies infections as in CF.

Common treatment strategies involve the use of iron chelators and quorum-sensing inhibitors to reduce iron availability, impair bacterial metabolisms, and prevent biofilm formation (Moreau-Marquis et al., 2009; Nair et al., 2020; Martin et al., 2021). Antibiotic treatment is prescribed once the pathogens are detected in sputum samples. However, detection of pathogens results more possibly from their dispersal from the biofilms, where antibiotic penetration and diffusion are restricted. Therefore, mucolytic agents and antibiotics are used in tandem to break mucus and biofilm matrix, and subsequently kill the embedded bacteria. Many mucolytic agents such as, alginate lyase enzyme AlyP1400, derived from a marine *Pseudoalteromonas* bacterium, have been reported for their promising antibiofilm activity (Daboor et al., 2021). Other hydrolases, including PslG and PelA, and Dispersin B were also found effective in reducing the viscoelastic properties of *P. aeruginosa* and *S. aureus* biofilms by targeting polysaccharide synthesis locus (psl), pellicle (pel) and polysaccharide intercellular adhesin (PIA), respectively (Yu et al., 2015; Baker et al., 2016; Suresh et al., 2019). The DNAse I therapy appears to further disrupt biofilms containing extracellular DNA (eDNA), which accounts for the cohesive nature of the biofilm matrix (Nair et al., 2020). And all these compounds seem to potentiate antibiotics against the infections. Still, the tools for quickly assessing the efficiency of these compounds vary from one experiment to the other, and their comparisons become even more challenging when multispecies biofilms are considered. This creates the need for new approaches to evaluate the effectiveness of the mucolytic agents by measuring the viscoelastic changes experienced by complex biofilms.

Here, we use pendant drop elastometry (PDE) to measure the rheological properties of single- and dual-species bacterial films, equivalent to early-stage biofilms. Biofilms commonly exhibit viscous (liquid-like) and elastic (solid-like) behaviors while dissipating and storing energy under an applied strain. Pendant drop elastomery generates an oscillatory deformation to record such energy in the form of a complex modulus corresponding to elastic and viscous properties of the biological films. We use this technique to examine the ability of disrupting agents, namely N-acetyl cysteine (NAC) and Cysteamine (CYST), to alter the mechanical properties of the interfacial films of *P. aeruginosa* and *S. aureus*. Previously, NAC and CYST were shown to disrupt biofilms and kill the embedded cells alone or in combination with antibiotics (Charrier et al., 2014; Li et al., 2020). We hypothesize that the mucolytic activity of NAC and CYST would be stronger when synergistic interactions occur among mucoid *P. aeruginosa* and *S. aureus* due to improved biofilm properties. We anticipate using PDE to measure the differences in the mechanical properties of composite films made by *P. aeruginosa* and *S. aureus* under competition or cooperation. Therefore, we investigate the interfacial properties of a mucoid strain of PAO1 (PAO1*mucA22*) and the wild-type PAO1 in combination with *S. aureus* (SH1000) to verify our hypothesis. Our results demonstrate that NAC and CYST significantly alter the micromechanics of single- and dual-species interfacial films of *P. aeruginosa* and *S. aureus*. The efficacy of the disrupting agents against these early-stage biofilms is quickly and reliably assessed using PDE.

## 2 Materials and methods

### 2.1 Microbial culture and growth conditions

The bacteria used in this study included wild-type *P. aeruginosa* (PAO1), mucoid *P. aeruginosa* (PAO1mucA22), and *S. aureus* (SH1000). Dispersing agents N-acetyl cysteine (NAC) and Cysteamine (CYST) were purchased from Fisher Scientific (Waltham, MA). The wild-type *P. aeruginosa* and *S. aureus* were cultured in a Lysogeny broth (LB) medium, containing 10 g/L tryptone, 5 g/L yeast extract, and 10 g/L NaCl suspended in Milli-Q water (Niepa et al., 2017). Mucoid *P. aeruginosa* cells were cultured in the LB medium without NaCl to maintain the mucoid phenotype. The cultures in the flasks were grown at 37°C with shaking at 200 rpm to obtain cells in the stationary phase after 18h. The growth curves were monitored by measuring optical density (OD) at 600nm every 10 min for 48 h using a plate reader (Cytation 5 imaging reader, BioTek Instruments Inc.) Biofilm experiments were performed by depositing 10 μL of bacterial suspension from overnight cultures on LB agar plates. The colonies were grown overnight at 37°C in static conditions.

### 2.2 Treatment with N-acetyl cysteine and cysteamine

To determine the drug concentration for achieving complete eradication, cells were treated with NAC and CYST at concentrations ranging from 0 to 10 mg/mL. Briefly, cells were grown in LB medium to reach the stationary phase and 5 μL aliquots were used to prepare subcultures in 96 well plates. The OD of cultures was recorded every 10 min for 48 h at 37°C using a plate reader (Cytation 5 imaging reader, BioTek Instruments Inc.) Complementary colony forming units (CFU) analysis was performed to determine cell survival after NAC and CYST exposure. Cells from the overnight cultures were washed thrice, followed by incubation with NAC and CYST at concentrations of 5 and 10 mg/mL for a duration of 6 h at 37°C. Following the incubation time, the treated cells were centrifuged, resuspended, and washed in 154 mM NaCl solution thrice to eliminate extracellular NAC or CYST and the cell debris. The number of cells that survived was then quantified by CFU of viable cells as previously described (Chen et al., 2003).

### 2.3 Electron microscopy

Scanning electron microscopy (SEM) was performed to analyze the architecture of PAO1, PAO1*mucA22*, and SH1000 cells treated with NAC and CYST. The cells were drop-casted on titanium coupons by depositing 5 μL of cell suspension to characterize the effects of disrupting agents on planktonic cells. To assess biofilm architecture, the colonies of the respective strains of bacteria were grown on LB agar plates and collected on titanium coupons using capillary peeling techniques (Yan et al., 2018). The samples were then fixed in 2.5% glutaraldehyde for 12 h at room temperature. Thereafter, samples were washed thrice in 154 mM NaCl solution, followed by a series of dehydrations in 30%, 50%, 70%, 90%, and 100% ethanol for 10 minutes each. To achieve complete drying while preserving cell morphology, the samples were dehydrated thrice in 100% ethanol prior to critical point drying with 100% ethanol and liquid CO_2_ in a Leica Balzer CPD030 (Leica, IL). Imaging was done with the Zeiss SIGMA VP electron microscope at an accelerating voltage of 5 kV.

### 2.4 Pendant drop elastometry and dilatational interfacial rheology

Interfacial films of PAO1, PAO1*mucA22*, and SH1000 cells were analyzed for the rheological properties after aging for 24h, and subsequently, after exposure to NAC and CYST for 6h. A cuvette was filled with 154 mM NaCl suspension of the relevant bacterial suspension. A microliter-sized droplet of hexadecane (20 μL) was formed and held at the tip of an inverted needle (14 gauge) attached to a syringe filled with hexadecane. The drop was aged in bacterial suspension for ~24 h. An image of the drop was captured on a CCD camera. The bacteria were allowed to adsorb at the clean interface of hexadecane and water for 24 h and the change in the interfacial tension (IFT) was recorded over time. During this adhesion, the drop shape was analyzed continuously, which provided solutions to the Young–Laplace equation, determining the interfacial tension. The dilatational interfacial properties of the bacterial film were analyzed by subjecting it to oscillatory sweep in small frequency ranges. Using the Attension Theta Pulsating Drop Module PD200 (Biolin Scientific, USA), disturbances corresponding to sinusoidal waves of the amplitude of ± 1 μL (5% strain) were introduced to the droplet to record and measure the dilatational viscoelasticity of the films at frequencies ranging from 0.1 to 1 Hz. The theoretical foundation for such measurements have been elucidated by Myrvold et al (Myrvold and Hansen, 1998). By oscillating the droplet and changing its surface area and shape at various frequencies, one can measure the Gibbs surface elasticity of the interfacial film according to the following relationship:


E=d γ/d ln A,

where the surface tension γ and the area A have a time dependence. This gives rise to a complex dilatational modulus described by:


E*=E’+i E”

where the storage modulus E’ and loss modulus E” describe the elastic contribution and the relaxation due to viscous dissipations in the film, respectively. Because the sinusoidal wave associated with the change of the surface area Δ lnA is proportional to exp (iωt), the complex modulus can be further described in terms of:


E*=|E|cos δ+i|E|sin δ,

where |E|= γ_a_/(A_a_/A_0_), to account for the interfacial tension (γ_a_) and surface area (A_a_) at the measured oscillation amplitude. The expression “*δ*” corresponds to a phase angle difference between the response of the interfacial tension and the droplet area due to the sinusoidal perturbation. Consequently, the complex modulus with a phase angle of 0° or 90° will correspond to purely elastic and purely viscous materials, while viscoelastic materials will have a phase angle ranging between 0° or 90°.

### 2.5 Statistical analysis

All the experiments were conducted at least in triplicate, and the standard errors shown. JMP14.1 Software (SAS Institute, NC) was used to conduct t-test and one-way ANOVA to establish the significance of the experimental conditions as a function of time for the IFT measurements, of frequency for the viscoelastic properties, and of concentration for the growth curves of CFU quantification. The aggregate size for the three microorganisms was compared as a function of drug treatment relative to the control. Differences with *p< 0.05* were considered statistically significant. The following notations “ns”, *, **, ***, and **** describe statistical differences, with *p* values corresponding to *p > 0.05*, *p< 0.05*, *p< 0.01*, *p< 0.001*, and *p< 0.0001*, respectively.

## 3 Results and discussion

### 3.1 Susceptibility of bacteria to N-acetyl cysteine and cysteamine

**Figure 1:**
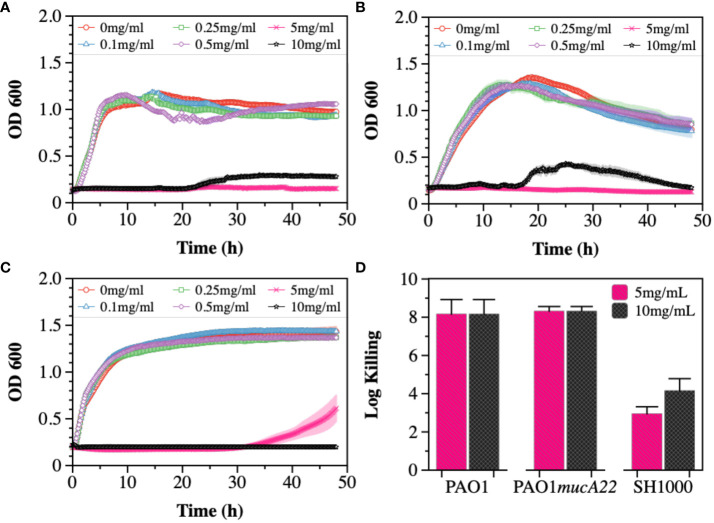
Susceptibility of wild type and mucoid *P. aeruginosa* PAO1 and PAO1*mucA22*, and *S. aureus* SH1000 to NAC. Growth curves were recorded while exposing **(A)** PAO1, **(B)** PAO1*mucA22* and **(C)** SH1000 to 0, 0.1, 0.25, 0.5, 1, 5 and 10 mg/mL NAC. The optical density at 600 nm (OD600) was measured every 10 minutes for 48 hours. **(D)** The killing activity of NAC on stationary phase cells was evaluated after treating the cells with concentrations of 5 and 10 mg/mL NAC for 6 h Log killing was determined by counting the number of viable cells on LB agar. No viable *P. aeruginosa* cells were detected, corresponding to cell eradication by 8 logs.

The susceptibility of PAO1, PAO1*mucA22*, and SH1000 cells to NAC, a precursor to antioxidant glutathione, was first tested (**Figure 1**). The antibacterial activity of NAC in CF patients has been associated with the drug’s ability to both degrade disulfide bridges in the biofilm EPS and increase oxidative stress for hindering protein biosynthesis (Zhao and Liu, 2010; Li et al., 2020). To measure its inhibitory activity against our model organisms, concentrations of 0.1, 0.25, 0.5, 1, 5 and 10 mg/mL NAC were added to subcultures of *P. aeruginosa* PAO1, PAO1*mucA22* and *S. aureus* SH1000 and the growth curves were recorded for 48 h at 37°C using the Cytation 5 plate reader (Biotek Inc.). Our results indicated dose-dependent inhibition of PAO1, PAO1*mucA22*, and SH1000 relative to the untreated controls. Specifically, the concentration of 5 mg/mL NAC completely suppressed the growth of all the species (**Figures 1A-C**), corroborating previous findings about the antibacterial activity of NAC (Li et al., 2020).

The activity of NAC against stationary phase cells was also assessed. The results indicated the effects of the compound to be more profound on *P. aeruginosa* PAO1 and PAO1*mucA22* strains, compared to *S. aureus* SH1000 (**Supplementary Figure 1**). Log killing was calculated by performing CFU analysis of the bacteria subjected to NAC treatment for 6 h. Bacterial exposure to 5 mg/mL NAC resulted in 8-log killing for both *P. aeruginosa* strains, as shown in **Figure 1D**. In contrast, 3-log and 4-log killing were observed for the SH1000 cells at concentrations of 5 mg/mL and 10 mg/mL, respectively (**Figure 1D**). Consequently, the concentration of 10 mg/mL NAC, which resulted in a significant reduction in cell growth and viability, was chosen to perform further studies.

**Figure 2:**
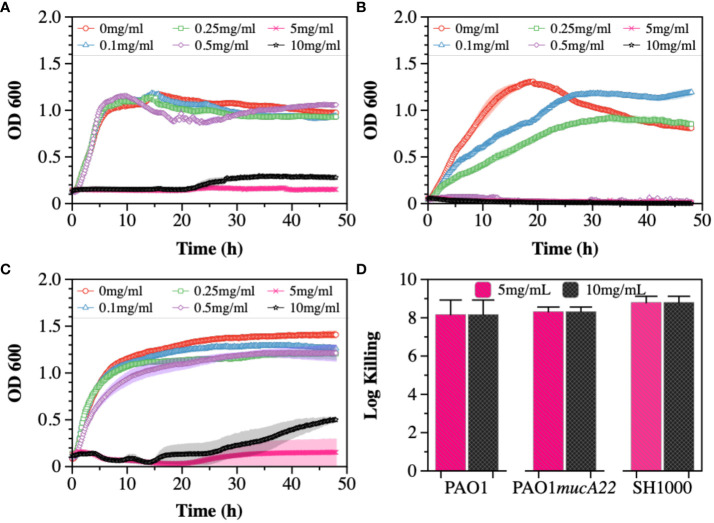
Susceptibility of wild type and mucoid *P. aeruginosa* and *S. aureus* to CYST. Growth curves were recorded while exposing **(A)** PAO1, **(B)** PAO1mucA22, and **(C)** SH1000 to 0, 0.1, 0.25, 0.5, 1, 5, and 10 mg/mL CYST. The optical density at 600 nm (OD600) was measured every 10 minutes for 48 hours. **(D)** The killing activity of CYST on stationary phase cells was evaluated after treating the cells with concentrations of 5 and 10 mg/mL CYST for 6 h. Log killing was determined by counting the number of viable cells on LB agar. No viable cells were detected, corresponding to cell eradication by 8 logs.

Cysteamine (CYST) was the second disrupting agent assessed in this study. The compound, commercially available under the trade name Lynovex® (Charrier et al., 2014), exhibits antimicrobial activity through increased levels of reactive oxygen species in the cell microenvironment (Fraser-Pitt and Mercer, 2021). Similar to the treatments with NAC, the susceptibility of PAO1, PAO1*mucA22*, and SH1000 to concentrations ranging from 0 to 10 mg/mL CYST was tested. The growth curves also indicated a dose-dependent inhibition of the strains (**Figures 2A-C**). Higher inhibitory activity against PAO1*mucA22* was recorded at concentrations as low as 0.5 mg/mL (**Figure 2B**). More interestingly, the treatment of stationary phase cells with 5 mg/mL CYST alone resulted in 8-log killing for all the strains, indicating a complete eradication of the bacteria (**Figure 2D**). Consequently, the concentration of 5 mg/mL CYST was chosen to perform further studies.

### 3.2 Effects of NAC and CYST exposure on PAO1, PAO1*mucA22*, and SH1000 cell morphology

**Figure 3:**
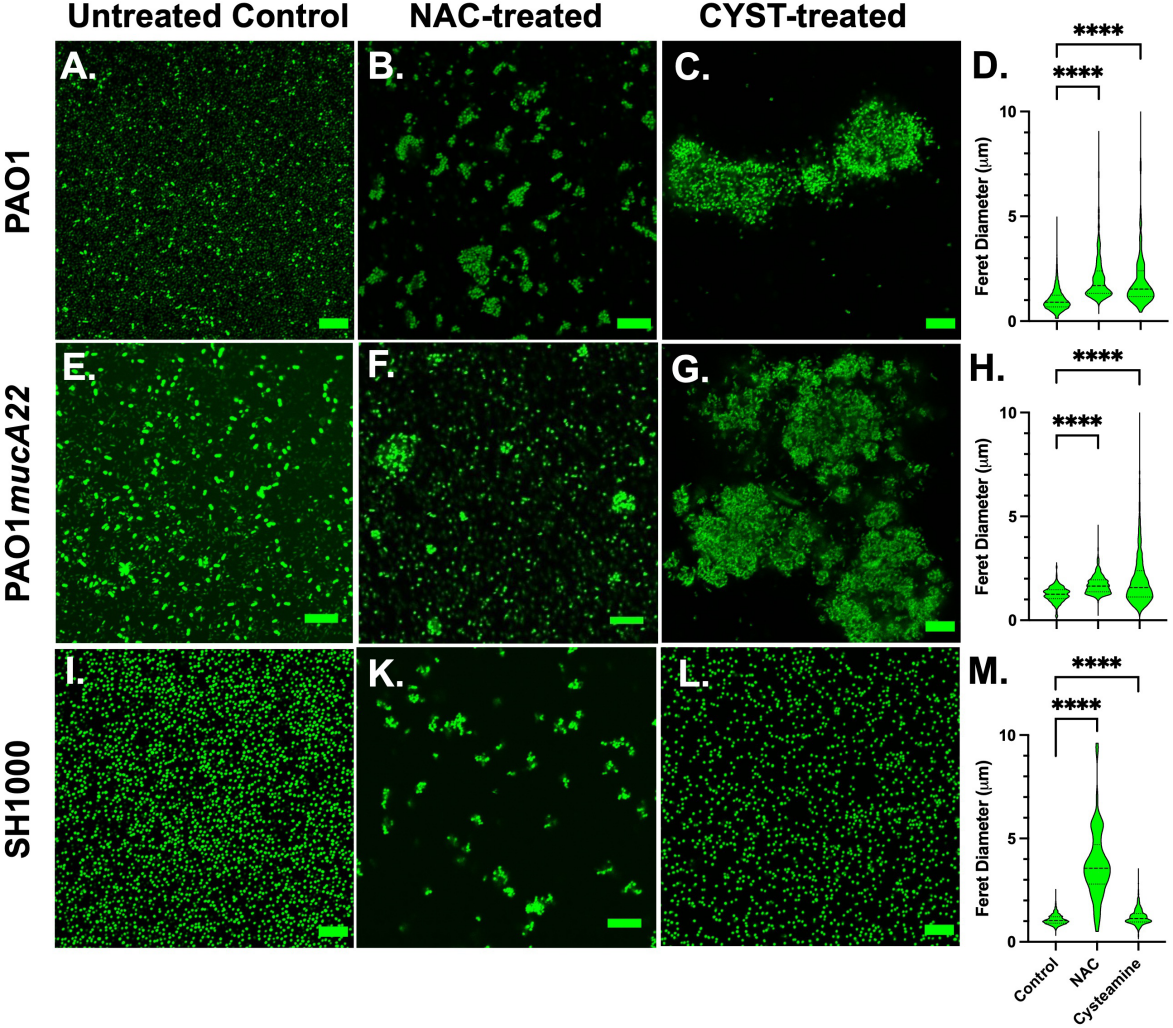
Confocal images of wild type and mucoid *P. aeruginosa* PAO1 and PAO1*mucA22* and *S. aureus* SH1000 following exposure to 10 mg/mL NAC and 5 mg/mL CYST for 6 hours. Confocal images of **(A)** control, **(B)** NAC-treated and **(C)** CYST-treated PAO1 cells reveal cell aggregation. **(D)** The Feret diameter for the aggregates is presented. The aggregate distribution for the **(E)** control, **(F)** NAC-treated, and **(G)** CYST-treated PAO1*mucA22* cells, as well as the corresponding **(H)** Feret diameter is also presented. Finally, aggregation of the **(I)** control, **(K)** NAC-treated, and **(L)** CYST-treated SH1000 cells and the corresponding **(M)** Feret diameter for each condition is presented. Statistical difference, corresponding to p< 0.0001 (****), is noted by comparing the treatments to the untreated control. SB: 10 μm.

Based on the screening results, CYST appeared more lethal to the three tested strains than NAC. The concentrations of 10 mg/mL NAC and 5 mg/mL CYST were selected to evaluate the effects of these disrupting agents on cell morphology and aggregation. Consequently, the cells were stained with Syto 9 following NAC and CYST treatment for 6 h and imaged through confocal microscopy to assess cell aggregation. Our results, shown in **Figure 3**, indicate that exposure of the two *P. aeruginosa* strains to NAC and CYST leads to the cells’ aggregation (**Figures 3A-H**). Cell clusters corresponding to a Feret diameter of 1-10 μm were observed, particularly in *P. aeruginosa* PAO1 and PAO1*mucA22* samples treated with CYST (**Figures 3C, G**). In contrast, cells in the control sample exhibited a smaller Feret diameter, between 1 and 5 μm (*p< 0.0001*) (**Figures 3D, H**).

Complementary analyses of NAC- and CYST-treated cells were performed using scanning electron microscopy to determine morphological changes on the cell surface and aggregation. Our results presented in **Supplementary Figure 1** indicate that for control *P. aeruginosa* PAO1 and PAO1*mucA22*, cell aggregation was associated with the secretion of EPS. EPS secretion was significantly reduced in the samples treated with NAC and CYST. Cell aggregation in the NAC- and CYST-treated samples was linked to physiological stresses through cell surface damage as seen in corresponding SEM images (**Supplementary Figure 1**).

The exposure of the SH1000 cells to NAC also resulted in cell aggregation (**Figure 3K**), corresponding to a Feret diameter largely distributed between 1-6 μm (**Figure 3M**). Such distribution was significantly higher than that obtained under control conditions or treatment with CYST (*p< 0.0001*). The latter condition, surprisingly, did not lead to major aggregation of the SH1000 cells (**Figures 3L, M**). Thus, the effects of the CYST differed on *P. aeruginosa* and *S. aureus* cells. The drug appears potent against all cells and leads to aggregation in *P. aeruginosa* but not in *S. aureus*. Only NAC causes aggregation of *S. aureus* SH1000 cells, and EPS secretions were not involved in the phenomenon (**Supplementary Figure 1**). The SEM analyses did not reveal any major morphological changes on the cell surface after the treatment, even when cell aggregation resulted (**Supplementary Figure 1**). These results suggest that NAC treatment induced molecular changes in *S. aureus*, possibly leading to the secretion of key surface adhesins (e.g., clumping factor A) and cell aggregation (Staats et al., 2023). In any case, NAC and CYST exhibit mucolytic activity that results in the disruption of intercellular interactions among cells and the disintegration and alteration of viscoelastic properties of the films.

### 3.3 Interfacial and viscoelastic properties of single-species films of PAO1, PAO1*mucA22*, and SH1000

**Figure 4:**
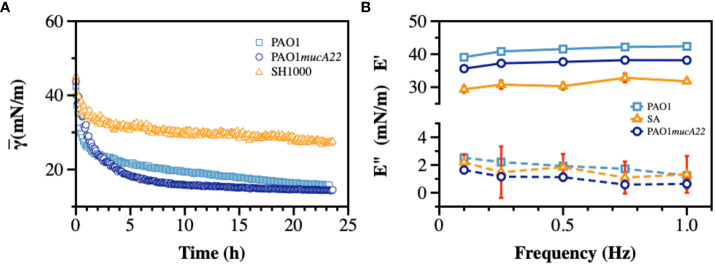
Interfacial and viscoelastic properties of monocultured bacterial films of wild type and mucoid *P. aeruginosa* and *S. aureus.* After an inverted hexadecane droplet was formed and held within the bacterial suspension, **(A)** The apparent IFT was measured over 24 h during biofilm development at the oil-water interface. At the end of 24 h, the droplet experienced sinusoidal waves to measure viscoelasticity and **(B)** the storage (E’) and loss (E”) moduli were recorded.

Dynamic IFT measurements were performed on monocultures of PAO1, PAO1*mucA22*, and SH1000 for 24 h to evaluate the adsorption profile of the bacteria at the hexadecane-water interface and the viscoelastic properties of the resulting films. As mentioned (Balmuri et al., 2020), the cells from overnight cultures were pelleted down and washed twice with 154 mM NaCl solution. The cell densities were adjusted based on the standard curves for each strain (**Supplementary Figure 2**), and the cell suspensions were exposed to hexadecane-water interfaces on a pendant drop for 24 h. The IFT decreased upon adsorption of cells to clean hexadecane-water interfaces. Our results showed minimal adsorption to hexadecane-water interface achieved by SH1000, compared to PAO1 and PAO1*mucA22*, as shown in **Figures 4A, B**, potentially due to the lack of EPS secretion by SH1000. Initially, at 54 mN/m, the IFT is reduced with the adsorption of PAO1 and PAO1*mucA22* to 15 mN/m and 14 mN/m, respectively (**Figure 4A**). On the other hand, in the presence of SH1000, the IFT reduced to 27 mN/m. The two *P. aeruginosa* strains reduced the interfacial tension to a greater extent, similar to the reported clinical isolates (Balmuri et al., 2020; Balmuri et al., 2022). This characteristic of the *P. aeruginosa* films is due to the adsorption of the cells at fluid interfaces and the secretion of EPS components to remodel the interfaces with viscoelastic films (Balmuri et al., 2022).

Therefore, the viscoelastic properties of the interfacial films formed by monocultures were characterized to evaluate their individual strength. To measure film viscoelasticity, the pendant drops were subjected to sinusoidal waves of ± 1 μL amplitude, corresponding to 5% strain, at a frequency ranging between 0.1–1 Hz (Myrvold and Hansen, 1998; Balmuri et al., 2020). Due to the weak adsorption of SH1000 cells to interfaces, we anticipated a lower strength for their corresponding films. *S. aureus* SH1000 indeed showed relatively weak interfacial films, with a storage modulus corresponding to 30 ± 1 mN/m (**Figure 4B**). On the other hand, interfacial films of the wild-type PAO1 and the mucoid PAO1*mucA22* cells exhibited a storage modulus of 41.2 ± 1.3 mN/m and 37.4 ± 1.1 mN/m, respectively (*p=0.0009* for all conditions). These results are consistent with our previous observations that mucoid films produce softer films, with a storage modulus significantly lower than that of non-mucoid films (Balmuri et al., 2020).

### 3.4 Effects of NAC and CYST on disruption of single-species films

**Figure 5:**
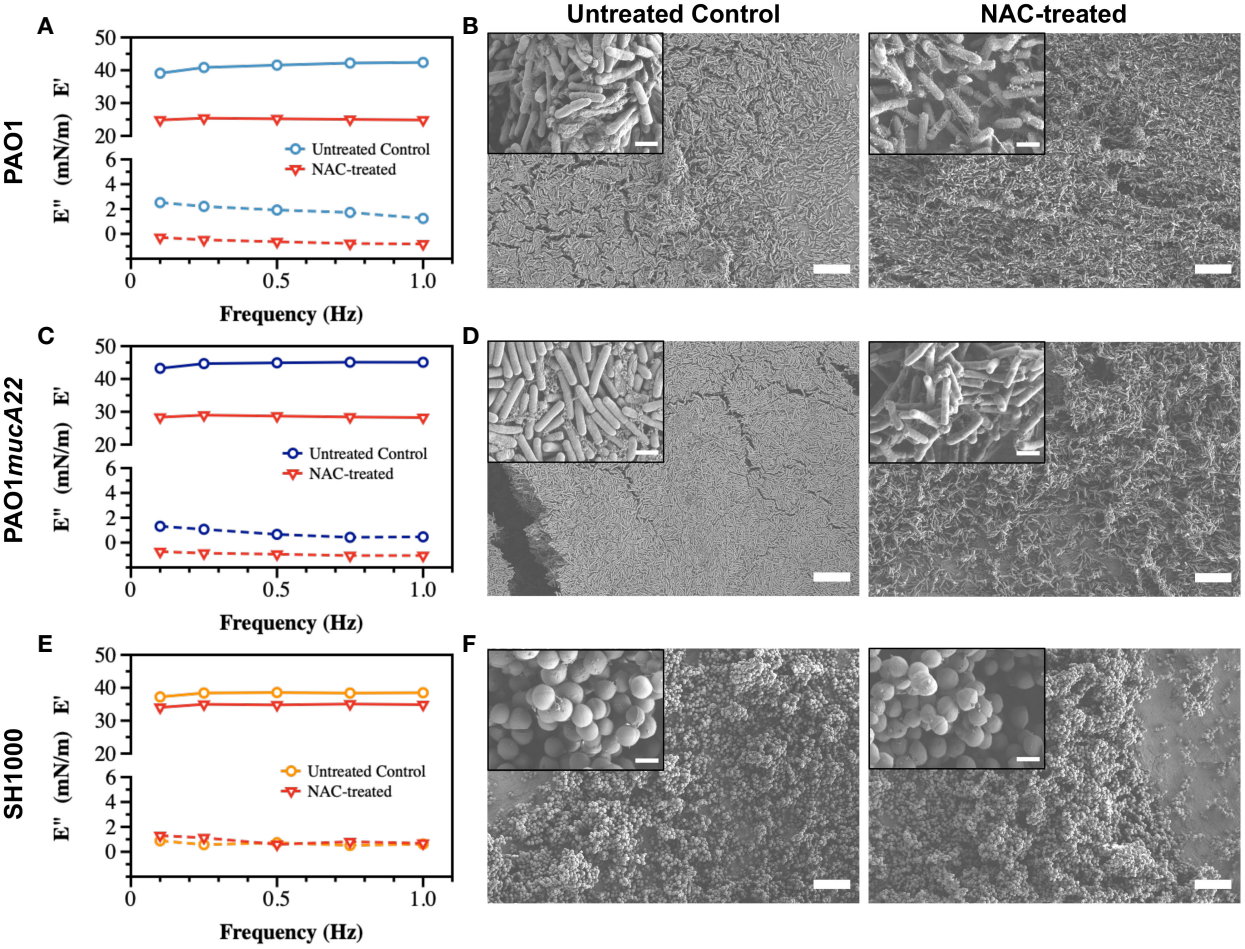
Viscoelastic properties and architecture of *P. aeruginosa* PAO1, *PAO1mucA22* and *S. aureus* SH1000 films. Storage and loss moduli of films of **(A)** PAO1, **(C)** PAO1*mucA22* and **(E)** SH1000 before and after 6 hours treatment with 10 mg/mL NAC are presented. Representative Scanning Electron Microscopy images of control and 10 mg/mL NAC-treated biofilms of **(B)** PAO1, **(D)** PAO1*mucA22* and **(F)** SH1000 are shown. SB: 10 μm (inset SB: 1 μm).

Pendant drop elastometry was performed to evaluate changes in the interfacial properties of films aged for 24 h. The viscoelastic properties of the interfacial films were first measured before the bacteria-laden interfaces were treated for 6 h with 10 mg/mL NAC or 5 mg/mL CYST carefully administered using a micropipette. The mechanical properties (the storage and the loss moduli) at the end of the treatment were again measured to evaluate the disruptive effects of each drug on the films. As shown in **Figure 5**, the viscoelastic properties of the interfacial films were significantly altered by the 6 h treatment with NAC. The storage modulus was expected to decrease because of the activity of the dispersing agent. As anticipated, the storage modulus of the interfacial films of PAO1 dropped from 41.2 ± 1.3 mN/m to 25.1 ± 0.2 mN/m, corresponding to a reduction of 39% (*p<0.0001*, **Figure 5A**).

To corroborate the changes in film viscoelasticity with the disintegration of film architecture, SEM was performed on single-species biofilms grown on LB agar for 24 h and treated with 10 mg/mL NAC. The biofilms were removed by capillary peeling and collected on the titanium coupons. The samples were further prepared for SEM imaging, as described above. Compared to control PAO1 biofilm supported by an EPS matrix (**Figure 5B**), the biofilm treated with NAC displayed a denatured matrix as shown in **Figure 5C**.

Similarly, the storage modulus of PAO1*mucA22* was substantially reduced by the NAC treatment, from 44.6 ± 0.8 mN/m to 28.5 ± 0.3 mN/m, corresponding to a 36% drop from its original modulus (*p<0.0001*, **Figure 5C**). While the control PAO1*mucA22* biofilms showed a thick and well-compacted biomass inherent to the mucoid *P. aeruginosa* biofilms (**Figure 5D**), the biofilm architecture was highly compromised upon treatment with NAC as shown in **Figure 5F**.

As for the *S. aureus* films, the SH1000-laden interfaces experienced a minimal reduction in their elastic property by 9.1% following NAC treatment, corresponding to a decrease in the storage modulus from 38.2 ± 0.6 mN/m to 34.7 ± 0.4 mN/m (*p<0.0001*, **Figure 5E**). Moreover, NAC treatment did not alter the integrity of SH1000 biofilm significantly. In fact, *S. aureus* SH1000 biofilm contained minimal EPS secretions (**Figure 5F**) as previously reported (Lamret et al., 2021). Overall, these results indicate that the effects of NAC are greater in *P. aeruginosa* strains than in *S. aureus* strains. This might also imply that NAC disrupts the biofilms by targeting the EPS components. Since the EPS secretions are more abundant in the *Pseudomonas* strains than in SH1000, the mucolytic activity of NAC is more pronounced in *P. aeruginosa* biofilms, correlating with a significant drop in the storage modulus of the corresponding interfacial films.

**Figure 6:**
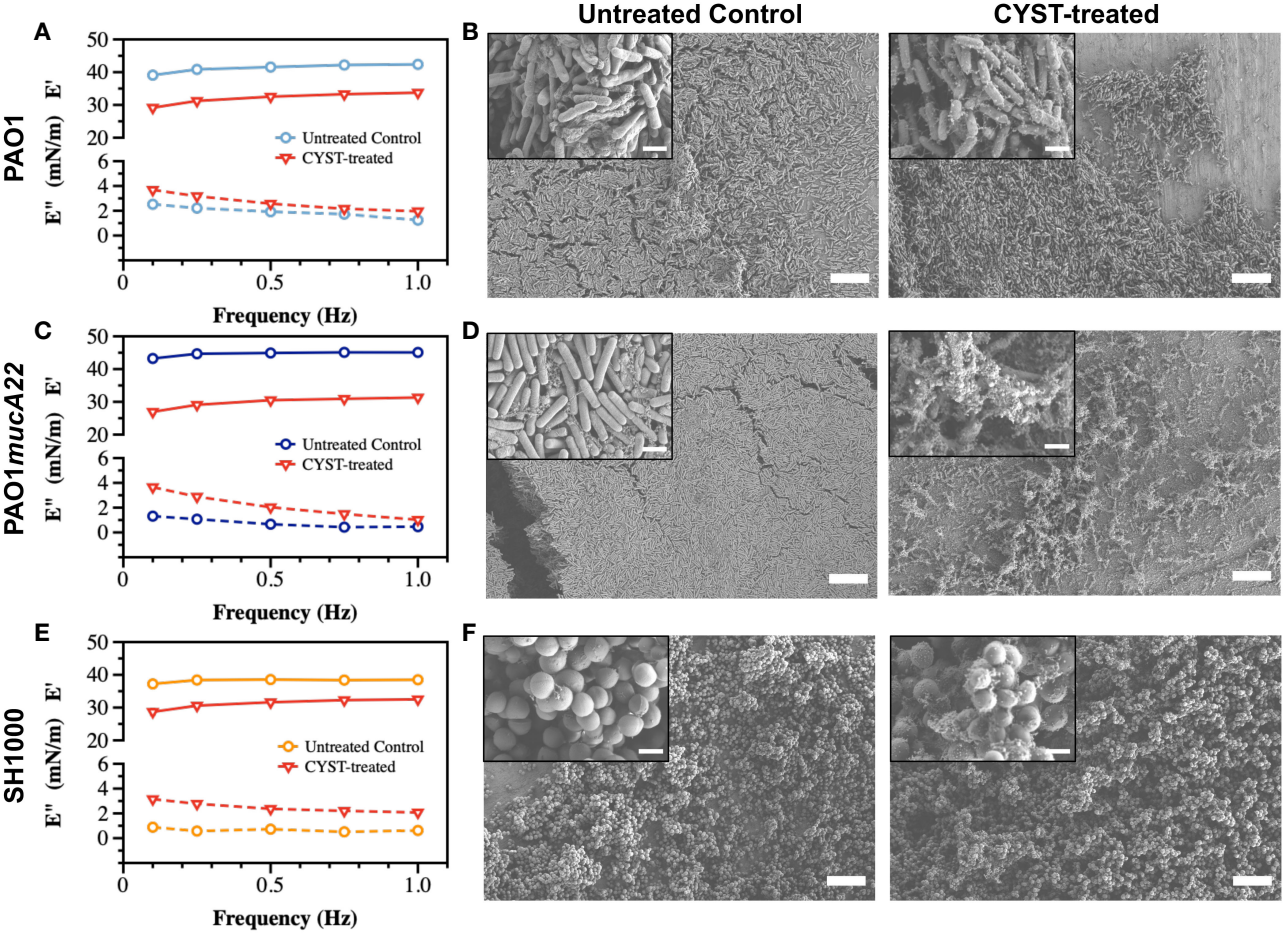
Viscoelastic properties and architecture of *P. aeruginosa* PAO1, PAO1mucA22 and *S. aureus* SH1000 films. The storage (E’) and loss (E”) moduli of films of **(A)** PAO1, **(C)** PAO1*mucA22* and **(E)** SH1000 before and after 6 h treatment with CYST are presented. Representative Scanning Electron Microscopy images of control and 10 mg/mL NAC treated biofilms of **(B)** PAO1, **(D)** PAO1*mucA22* and **(F)** SH1000 are shown. SB: 10 μm (inset SB: 1 μm). For the sake of comparison, storage and loss moduli, and SEM images of the controls are reproduced.

Similarly, films of PAO1, PAO1*mucA22* and SH1000 at fluid interfaces experienced viscoelastic changes due to CYST exposure for 6 h (**Figure 6**). The storage modulus of interfacial films of PAO1 cells decreased from 41.2 ± 1.3 mN/m to 32.0 ± 1.8 mN/m, corresponding to a 24% drop of its elasticity (*p<0.0001*, **Figure 6A**). Qualitatively, such reduction in the elastic modulus of the PAO1 films through CYST translated into the denaturation of its biofilm with a clear disruption of the EPS matrix (**Figure 6B**). However, the effects of CYST were more pronounced on the mucoid *P. aeruginosa* (**Figure 6C**). The PAO1*mucA22* films became unstable during the 6 h of CYST exposure. The sudden disruption of the EPS matrix, as illustrated by the SEM analysis (**Figure 6D**), resulted in a drastic change in the interfacial properties of the films, leading to the removal of the pendant drop. Consequently, the storage modulus could be recorded only after 3 h, while the pendant drop was still stable (**Supplementary Figure 6**). Then, the elastic property of the interfacial films of PAO1*mucA22* was reduced by 32%, corresponding to a change in storage modulus from 44.6 ± 0.8 mN/m to 29.8 ± 1.8 mN/m (*p<0.0001*, **Figure 6C**).

CYST appeared more disruptive than NAC against *S. aureus* films, even if the reduction in the viscoelastic properties of the SH1000 films was minor, compared to the *P. aeruginosa* films. CYST treatment for 6 h reduced the storage modulus of the SH1000 films by 18.4%, from 38.2 ± 0.6 mN/m to 31.1 ± 1.6 mN/m (*p<0.0001*, **Figure 6E**). From the SEM analyses, CYST appeared to induce some oxidative stress on the cell surface, with no major dispersal of the biofilm matrix (**Figure 6F**). Overall, these results indicate that CYST alters the interfacial properties of *P. aeruginosa* more profoundly than *S. aureus*.

### 3.5 Viscoelastic properties of mixed interfacial films of *S. aureus* and *P. aeruginosa*

**Figure 7:**
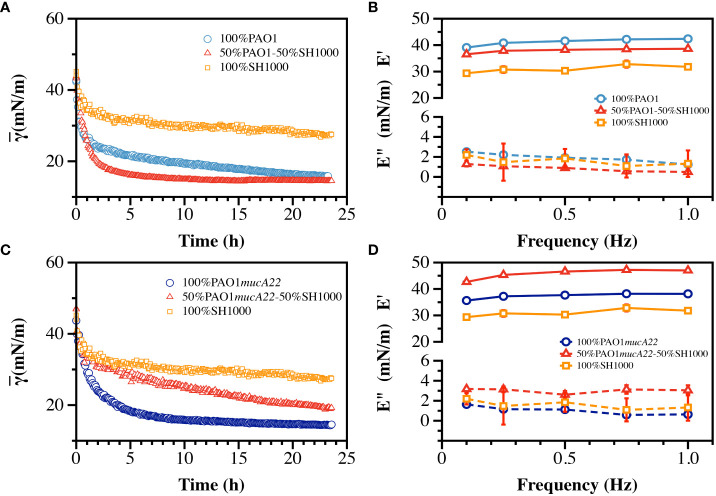
Interfacial and viscoelastic properties of monocultured and cocultured films of wild type and mucoid *P. aeruginosa* and *S. aureus.* **(A)** The dynamic IFT for 24 h and **(B)** the storage (E’) and loss (E”) moduli of PAO1, SH1000 and their mixture at equal cell ratio were recorded after film formation at the hexadecane-water interface. Similarly, **(C)** the dynamic IFT for 24 h and **(D)** the storage (E’) and loss (E”) moduli of PAO1*mucA22* and SH1000 and their mixture at equal cell ratio were recorded after film formation at the hexadecane-water interface.

The ability of *S. aureus* and *P. aeruginosa* to remodel fluid interface simultaneously was investigated. The competition among the two species is well documented (Limoli et al., 2017; Price et al., 2020). Thus, to assess the potential impact of an antagonistic bacterial interaction on film viscoelasticity (**Supplementary Figure 3**), pendant drops of hexadecane were held for 24 h in suspensions of PAO1 and SH1000 mixed at equal cell count. The cells adsorbed at the oil-water interface to build dual-species films, which correlated with a reduction of the apparent interfacial tension over 24 h (**Figure 7A**). The rate of adsorption of cells in the mixed suspensions was higher than that in PAO1 suspensions alone, and even more relative to the condition with SH1000 cells alone. SH1000 cells reduced the hexadecane-water IFT from 54 mN/m to 27 mN/m, while PAO1 cells reduced it to 15 mN/m. On the other hand, the 50% PAO1-50% SH1000 mixture reduced the hexadecane-water IFT to 14 mN/m (**Figure 7A**).

To understand how the high IFT reduction recorded with the formation of the dual-species films correlated with its mechanical strength, the viscoelasticity of the interfacial films was measured (**Supplementary Figures 4, 5**). Interestingly, the storage modulus of the mixed films was weaker (37.9 ± 0.8 mN/m) than that of PAO1 alone (41.2 ± 1.3 mN/m), indicating that the faster interfacial remodeling in the dual-species condition was associated with poor quality films (**Figure 7B**). Still, the mixed films were stronger than those of SH1000, which exhibited a storage modulus corresponding to 31.0 ± 1.3 mN/m (*p<0.0001*, **Figure 7B**). The results might suggest that the mechanical strength of the dual-species films might be strongly dominated by PAO1 remodeling activity at the interface.

To comprehend the impact of a potentially synergistic interaction between *S. aureus* and *P. aeruginosa*, SH1000 and PAO1*mucA22* suspensions were mixed at equal ratio and the cells were allowed to adsorb at the hexadecane-water interface. Under this condition, the cells in the mixtures adsorb and remodel the interfaces to an extent that is lesser than when PAO1*mucA22* and even PAO1 alone. The apparent IFT of the interface exposed to PAO1*mucA22* alone reduced at a faster pace to 14 mN/m, while that of the mixture with PAO1*mucA22* and SH1000 slowly decreased to 19 mN/m (**Figure 7C**).

Surprisingly, the results of the mechanical testing showed that the viscoelastic properties of the dual-species films are stronger when PAO1*mucA22* and SH1000 remodel the interfaces altogether. While PAO1*mucA22* cells alone built films with a storage modulus of 37.4 ± 1.1 mN/m, they remodeled the hexadecane-water interfaces with a stronger film (45.8 ± 1.9 mN/m) in the presence of SH1000 (*p<0.0001*, **Figure 7D**). These results corroborate previous findings that films of mucoid *P. aeruginosa* are softer than those of their nonmucoid counterparts (Balmuri et al., 2020). However, the ability of the mucoid *P. aeruginosa* to build the strongest films in the presence of SH1000 is surprising and might indicate a cooperative behavior between the PAO1*mucA22* and SH1000 strains. Such cooperation might improve the mechanical properties of the resulting biofilms, which could affect disease outcome negatively. For instance, films of the mucoid *P. aeruginosa* and *S. aureus* might be more resilient to removal through mechanical force than films with non-mucoid *P. aeruginosa*.

### 3.6 Effects of NAC and CYST on disruption of dual-species films

**Figure 8:**
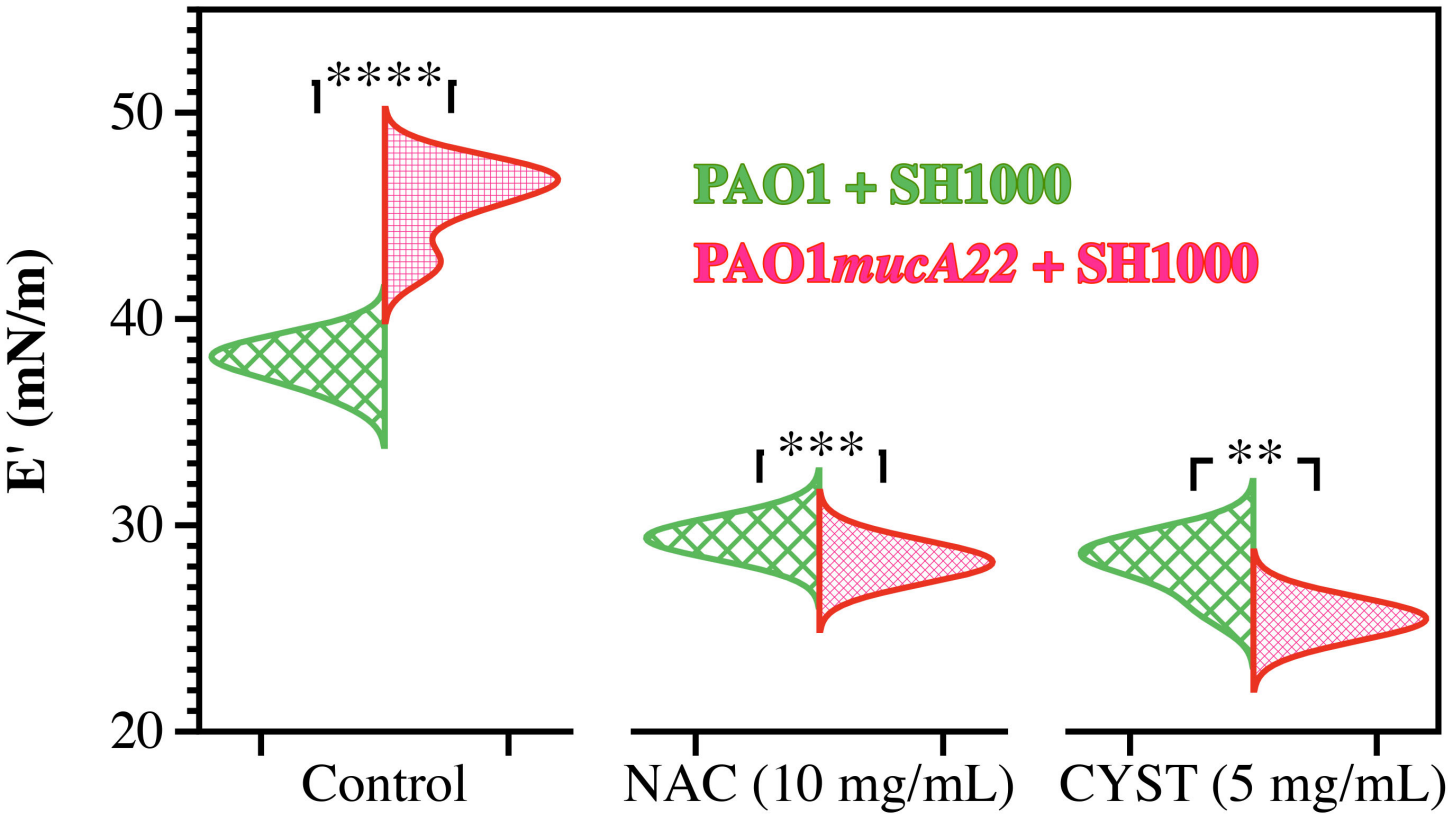
Elastic properties of composite films of *P. aeruginosa* with *S. aureus* before and after treatment with NAC and CYST. Only the storage (E’) moduli of composite films of PAO1 + SH1000 cells, and PAO1*mucA22* + SH1000 cells are presented. Statistical significance is noted regarding the difference in the elastic properties of the composite films with PAO1 and PAO1*mucA22* before and after treatments with NAC and CYST. Statistical differences corresponding to p< 0.01, p<0.001, and p< 0.0001, are noted **, ***, and ****, respectively.

The dual-species films were exposed to NAC and CYST to investigate how these disrupting agents alter the rheological properties of *P. aeruginosa* and *S. aureus*. The films were formed on a pendant drop exposed to an equal ratio of *P. aeruginosa* and *S. aureus* for 24 h. They were subsequently treated with 5 mg/mL CYST and 10 mg/mL NAC for 6 h. Before and after the drug treatment, the viscoelastic properties of the dual-species films were measured. Our results demonstrated that the drug significantly weakened the elastic properties of the films, and the effects are more potent on dual-species films with mucoid *P. aeruginosa* (*p<0.01* for all conditions vs. controls, **Figure 8**). As stated, mixtures of PAO1*mucA22* and SH1000 remodel fluid interfaces with a robust composite film, displaying a storage modulus of 45.8 ± 1.9 mN/m. However, the exposure to NAC and CYST reduced the film elasticity to 28.2 ± 0.4 mN/m and 25.4 ± 0.4 mN/m, respectively (*p<0.0001* for treatment conditions vs. control). The exposure of the composite films of PAO1 and SH1000 to NAC and CYST altered the elastic properties of the films similarly. Initially at 37.9 ± 0.8 mN/m, the storage modulus of the films decreased to 29.4 ± 0.3 mN/m and 28.1 ± 1.3 mN/m upon treatment with 10 mg/mL NAC and 5 mg/mL CYST, respectively (*p<0.0001* for treatment conditions vs. control). From these tests, CYST appeared more potent than NAC, as a lower dose of CYST was required to cause a more substantial disruption of the film matrix. Still, the ability of both mucolytic agents to reduce the mechanical properties of the interfacial was clearly established by these viscoelastic measurements.

To correlate the change in the rheological properties of the composite films and their architectures, the biofilms formed with mixtures of *P. aeruginosa* and *S. aureus* were also treated with 5 mg/mL NAC and 10mg/mL CYST. The films were further characterized through SEM.

**Figure 9:**
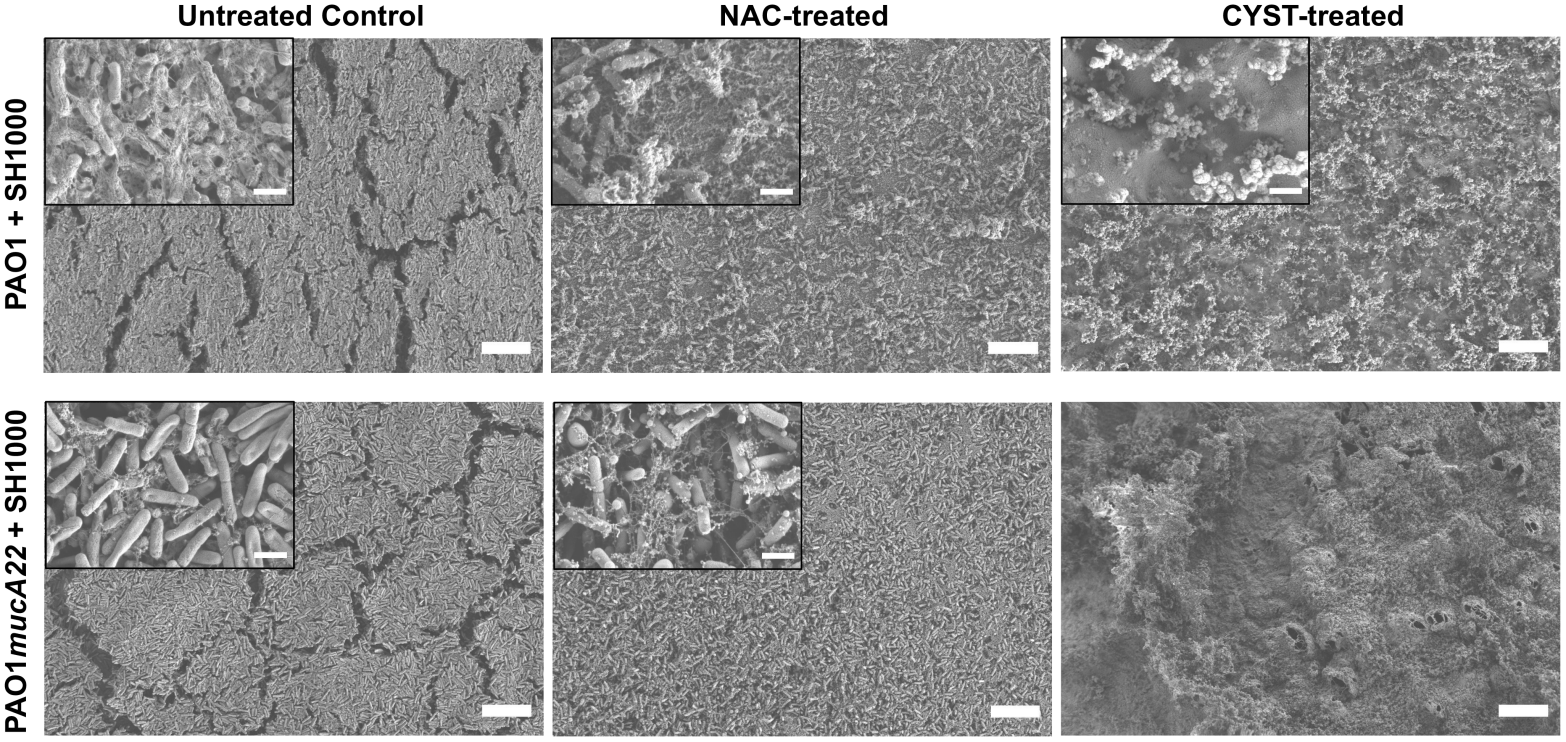
Change in the architectures of mixed biofilms of *P. aeruginosa* with *S. aureus* after treatment with NAC and CYST. Scanning electron microscopy was performed on biofilms of PAO1 + SH1000 cells, and PAO1mucA22 + SH1000 treated with 10 mg/mL NAC and 5 mg/mL CYST. SB: 1 mm (inset SB: 100 nm).

The SEM micrographs in **Figure 9** reveal a loss of biofilm cohesiveness and film integrity in all the treatment conditions (**Figure 9**). Compared to the untreated control, loose and very randomized cell packing resulted from treating the PAO1-SH1000 composite film with NAC and CYST. The drug treatment also denatured the PAO1*mucA22*-SH1000 composite films. More debris of EPS was observed on the samples treated with NAC, while those treated with CYST displayed air pockets. Because of the weak mechanical strength, the samples treated with CYST could not be imaged at higher magnification without charging the samples (**Figure 9**). These results indicated that the higher reduction of film elasticity, measured using the pendant drop elastometry, is associated with the disintegration of the film architecture due to the mucolytic drug activity.

## 4 Conclusions

Our results show that disruptive agents such as NAC and CYST tested in this study substantially inhibited the growth of monocultured PAO1, PAO1*mucA22*, and SH1000, as evidenced by growth curve analyses, biofilm experiments, and electron microscopy analyses. NAC and CYST treatments showed more than 30% disruption of interfacial films of monocultured PAO1, PAO1*mucA22*, and SH1000 films. It is also evident that the disruptive agents tested in this study are more effective against *P. aeruginosa* strains than *S. aureus*. Cocultured SH1000 with wild-type PAO1 showed reduced film strength, possibly due to the competition between them as reported by others. Interestingly, cocultures of SH1000 with mucoid PAO1*mucA22* showed increased strength of their interfacial films, possibly due to their cooperative behavior. SEM analyses of the biofilms also evidenced disruption upon treatments with NAC and CYST. Our results corroborated prior studies which showed competition between the wild type PAO1 and SH1000 and the co-existence of mucoid PAO1*mucA22* with SH1000. NAC and CYST treatments were also effective in disrupting the interfacial films formed by cocultures of SH1000 with wild-type PAO1 and mucoid PAO1*mucA22*. Overall, our system provides a sensitive platform for measuring the disruption abilities of chemical compounds.

## Data availability statement

The original contributions presented in the study are included in the article/**Supplementary Material**. Further inquiries can be directed to the corresponding author.

## Author contributions

SB: Conceptualization, Data curation, Formal Analysis, Investigation, Methodology, Writing – original draft, Writing – review & editing. SN: Conceptualization, Data curation, Formal Analysis, Investigation, Methodology, Writing – original draft. HU: Conceptualization, Data curation, Formal Analysis, Investigation, Methodology, Writing – original draft, Writing – review & editing. TN: Conceptualization, Data curation, Formal Analysis, Funding acquisition, Investigation, Methodology, Project administration, Supervision, Writing – original draft, Writing – review & editing.
