# Supplementary material for: Altering the interfacial rheology of Pseudomonas aeruginosa and Staphylococcus aureus with N-acetyl cysteine and cysteamine
Source: Front Cell Infect Microbiol. 2024 Jan 16;13:1338477. doi: 10.3389/fcimb.2023.1338477 (PMC10834029; doi:10.3389/fcimb.2023.1338477)
Supplement: Supplementary file 1 [file DataSheet_1.pdf]

Supplementary Material for

Altering the Interfacial Rheology of *Pseudomonas aeruginosa* and *Staphylococcus aureus* with N-acetyl cysteine and Cysteamine

*Sricharani Rao Balmuri[1], Sena Noaman[1], Huda Usman[1], Tagbo H. R. Niepa[1,2,3,4,5]**

[1]Department of Chemical and Petroleum Engineering, [2]Center for Medicine and the Microbiome, [3]The McGowan Institute for Regenerative Medicine, University of Pittsburgh, Pittsburgh, PA, United States.

[4]Department of Chemical Engineering, [5]Department of Biomedical Engineering, Carnegie Mellon University, Pittsburgh, PA, United States.

*Corresponding author: tniepa@andrew.cmu.edu

Keywords: *Pseudomonas aeruginosa, Staphylococcus aureus, fluid interfaces, thin-film, interfacial tension (IFT), viscoelastic materials, N-acetyl cysteine, Cysteamine.*

This supporting information includes: 1) the SEM analyses for the effects of 10 mg/mL NAC and 5mg/mL CYST exposure on PAO1, PAO1*mucA22,* and SH1000 morphology; 2) the standard curves relating Colony-forming units to an optical density at 600 nm; 3) PAO1+SH1000 and PAO1*mucA22*+SH1000 co-culture on agar plates for pyocyanin production; 4) Pendant drop elastometry for full compression of composite films; and 5) Dynamic interfacial tension measurement in the presence of NAC and CYST

1. Effects of NAC and CYST exposure on cell morphology

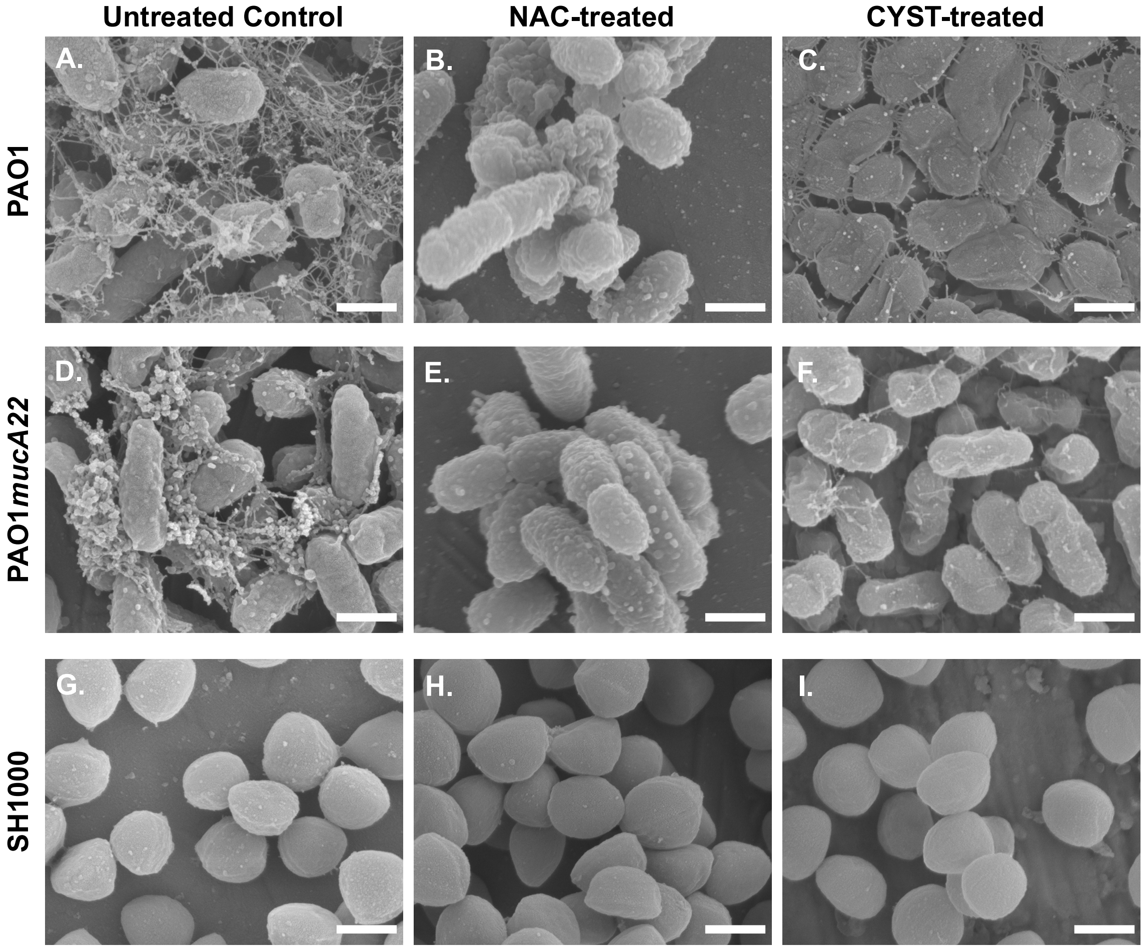

Figure S1. SEM images of planktonic bacteria treated with NAC and CYST. (A-C) represent the control, NAC-treated and CYST-treated cells of PAO1. (D-F) represent the control, NAC-treated and CYST-treated cells of PAO1mucA22. (G-I) represent the control, NAC-treated and CYST-treated cells of SH1000.Scanning electron microscopy was performed after 6 h drug treatment with NAC and CYST at the concentrations of 10 mg/mL. SB: 1μm.

To understand the effects of NAC and CYST on the cell morphology, PAO1, PAO1*mucA22,* and SH1000 were grown overnight and treated with 10 mg/mL NAC or 5 mg/mL CYST for 6 h. The samples were prepared for SEM and imaged using the Zeiss SIGMA VP electron microscope at an accelerating voltage of 5 kV. NAC and CYST have a stronger impact on *P. aeruginosa* cells, altering cell morphology, compared to *S. aureus* cells.

2. Calibration curves of PAO1, PAO1*mucA22* and SH1000

Colony-forming units (CFUs) corresponding to various optical densities ranging from OD 600 of 0–1 were determined. The optical density was measured for 5 different cell suspensions using a spectrophotometer (ThermoFisher Scientific, USA) in four replicates. A standard curve was established and employed to determine the necessary densities for the experiments conducted in this study.

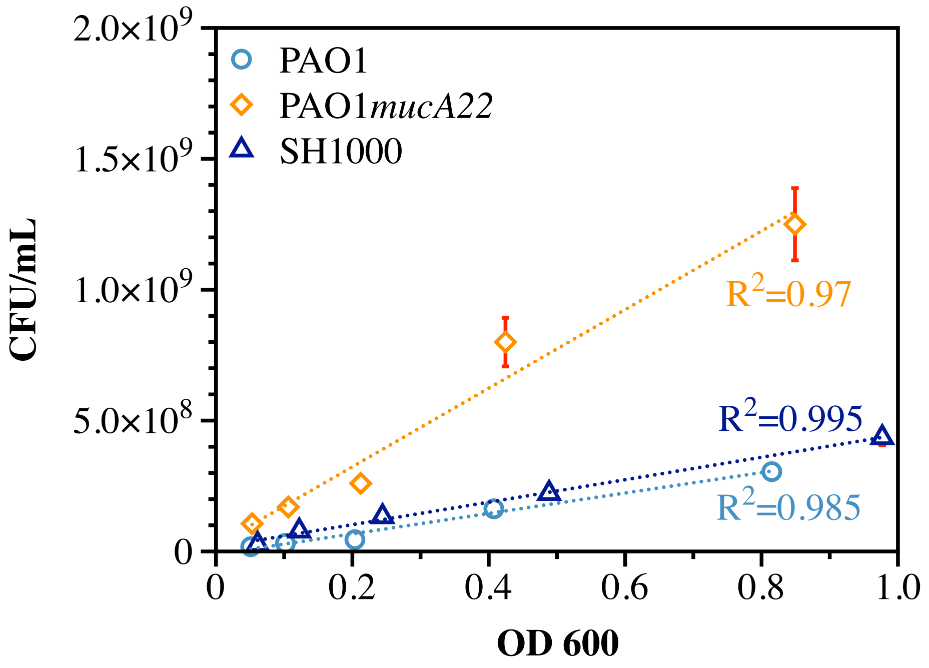

Figure S2. Standard curves of PAO1, PAO1*mucA22* and SH1000. Each point represents the mean of standard error of four samples.

3. Cocultures of *S. aureus* in the presence of wild type and mucoid *P. aeruginosa*.

SH1000 was grown in the presence of PAO1 and PAO1*mucA22* to analyze how the cooperative and competitive behaviors of the strains alter the mechanical properties of the resulting biofilms. Briefly, 10 mL of the respective overnight cultures were deposited in the center of the Petri dish prepared with 0.5% LB agar. The cells were grown overnight on the agar plates and digital images were recorded to qualitatively determine the differences in growth (**Fig. S3**). Colonies for the

cocultured conditions were formed by adding 5 mL of overnight cultures from both the strains (*P. aeruginosa* and *S. aureus*) on the Petri dishes. Bacteria formed pellicles with differences in their macroscopic morphologies. PAO1 showed increased secretion of the green pigment as shown in the first panel to the left. The green pigment is most likely expected to be pyocyanin as reported by many studies in the literature. [1-3] Pyocyanin is one of the many virulence factors produced by wild type *P. aeruginosa* to combat *S. aureus*. However, mucoid *P. aeruginosa* are not known to produce this compound. Our results also indicate a similar observation as no major difference was observed in the cocultures of PAO1*mucA22* and *S. aureus*.

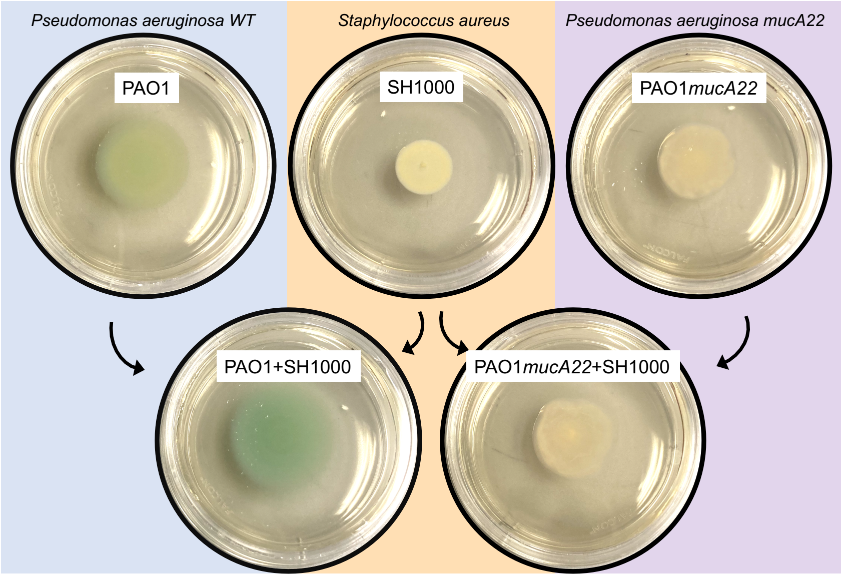

Figure S3. Overnight growth in petri dishes of monocultures and cocultures of S. aureus SH1000 and P. aeruginosa PAO1 and PAO1mucA22. Cocultures consisted of SH1000 in the presence of PAO1 exhibit pyocyanin secretion in response of competition, unlike mixed biofilm SH1000 and PAO1mucA22 where cooperation occurs.

4. Pendant drop tensiometry

Drop shape profiles of single- and dual-species bacterial films subjected to compression. As mentioned previously, bacteria are allowed to adsorb to the clean oil-water interfaces for a duration

of 24 hours, forming an interfacial bacterial film. The top panel shows a representative interfacial film of wild-type PAO1 subjected to various stages of compression. A robust film was formed by PAO1 cells as shown in the viscoelastic measurements. Representative compression images of the cocultured films of PAO1 and SH1000 cells are shown in **Figure S4**. A strong film was formed, corroborating with the viscoelastic measurements shown in **Figure 7**. On the other hand, SH1000 cells failed to form an interfacial bacterial film evident from the lack of film formation under compression as shown in the bottom panel.

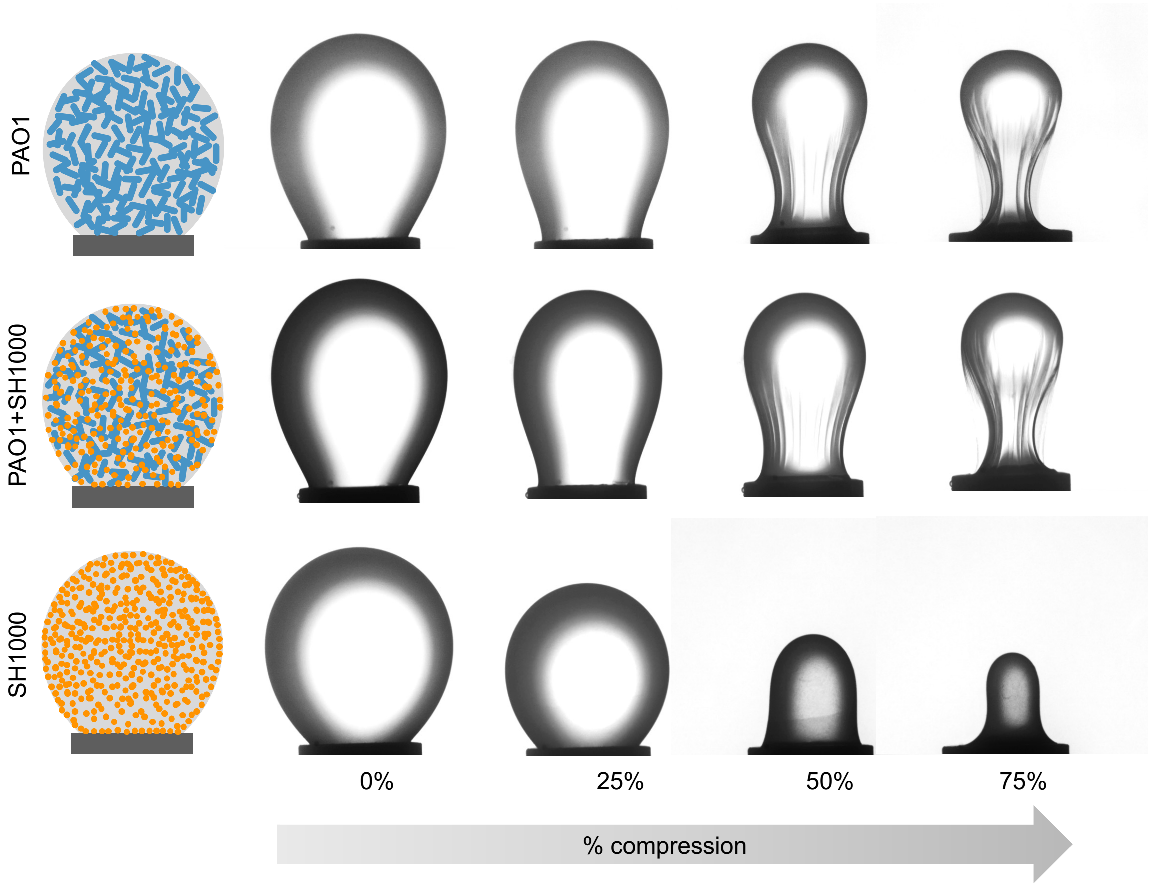

Figure S4. Representative images of droplet compression encompassing the films formed by monocultured PAO1, cocultured SH1000 and monocultured SH1000 cells.

Similarly, the film compression pattern was compared between the mucoid PAO1*mucA22* cells and the SH1000 cells (**Fig. S5**). The top panel shows a representative interfacial film of

PAO1*mucA22* subjected to various stages of compression. A robust film was formed by PAO1 cells as shown in the viscoelastic measurements. Representative compression images of the cocultured films of PAO1*mucA22* and SH1000 cells are shown in **Figure S5**. A strong film was formed in the presence of PAO1*mucA22,* corroborating with the viscoelastic measurements shown in **Figure 7**.

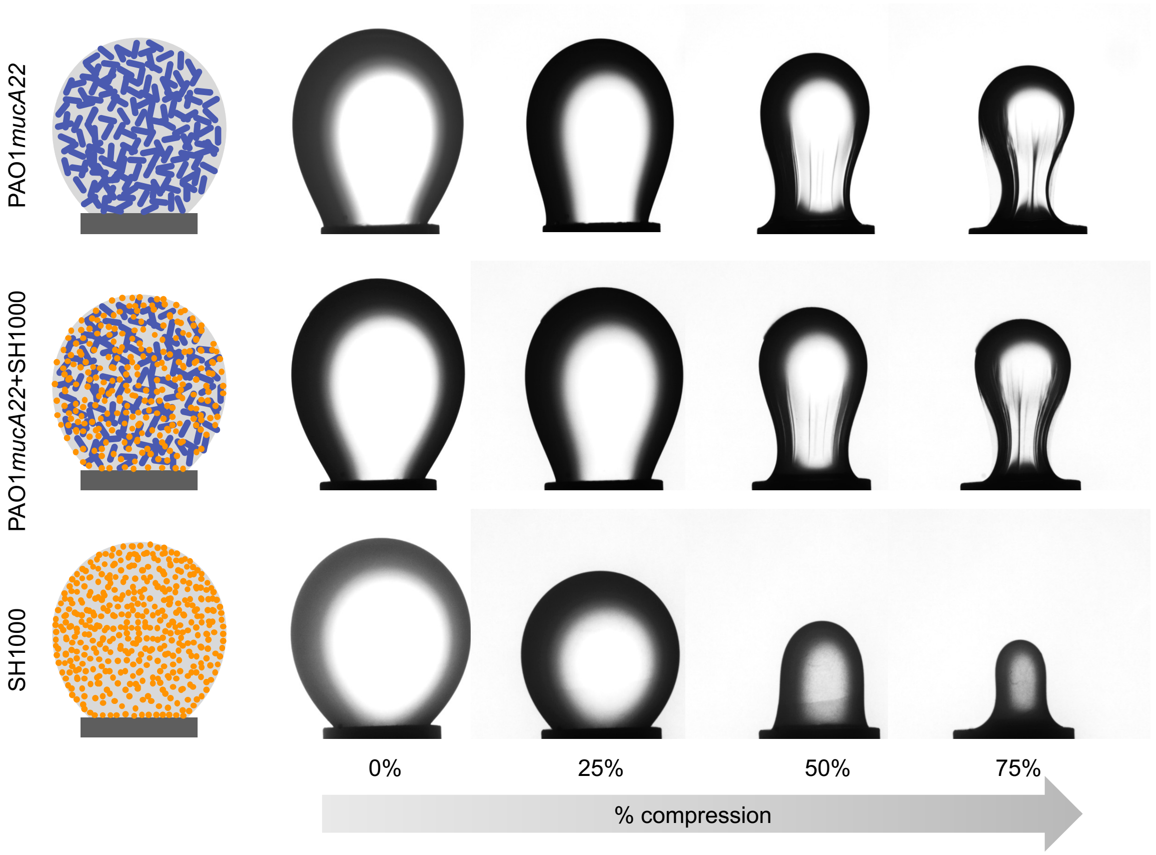

Figure S5. Representative images of droplet compression encompassing the films formed by monocultured PAO1*mucA22*, cocultured SH1000 and monocultured SH1000 cells. Compression of the SH1000 pendant drop is reproduced for a sake of comparison.

5. Effect of disrupting agents on interfacial properties

Pendant drop tensiometry was performed to evaluate the interfacial properties of films followed by drug treatment with each of the disruptive agents NAC and CYST. Cells were allowed to adsorb to the clean hexadecane-water interface for a duration of 24 h forming interfacial bacterial films.

After the duration of 24 h, NAC or CYST were carefully administered to the bacterial suspension via micropipette. The pendant droplets were rendered highly unstable with the addition of 10 mg/mL CYST, probably due to disruption of interfacial films. Therefore, the concentration of CYST was reduced to 5 mg/mL for pendant drop experiments. The mechanical properties following a 6-hour drug treatment were compared with those observed at the end of the original 24-hour period with untreated interfacial films. This comparison aimed to assess the disruptive effects of each drug on bacterial films. **Figure S6 (A-C)** depicts the change in interfacial properties after the addition of 10 mg/mL NAC to PAO1, PAO1*mucA22,* and SH1000 interfacial films. Dynamic interfacial tension (γ) during the adsorption of cells was plotted in addition to the changes in IFT after introducing the disruptive agents. The change in IFT following the addition of NAC and CYST is shown in red (**Fig. S6**). The introduction of 10 mg/mL NAC to PAO1 films did not show a significant reduction in the equilibrium IFT, shifting from 12.88 mN/m to 12.13 mN/m. Conversely, PAO1*mucA22* exhibited a noticeable decrease in IFT, from 17.81 mN/m to 16.36 mN/m upon NAC addition (**Fig. S6 B**). Additionally, the treatment of SH1000 interfacial films with 10 mg/mL NAC resulted in a decrease in IFT from 17.14 mN/m to 15.54 mN/m within 6-hours.

Conversely, introducing CYST to all three monocultures—PAO1, PAO1*mucA22*, and SH1000 after 24 hours—resulted in a sudden decrease in IFT. This abrupt shift could be attributed to alterations in droplet shape following the addition of CYST, as illustrated in **(Fig S6 D-F).** This behavior contrasts with the impact of NAC, likely owing to the inherent surface activity of CYST itself. As expected, the integrity of the interfacial films was compromised due to the disruptive nature of these agents.

These observations were consistent with our scanning electron microscopy results where NAC exhibited minimal effects on cell morphology in comparison with PAO1 and PAO1*mucA22* **(Fig 5 & 6)**. Finally, PAO1*mucA22* films could remain stable for the entire duration of the 6 h treatment period therefore the storage modulus was recorded after the 3 h treatment period. Interfacial films of PAO1*mucA22* showed a reduction in storage modulus by 32% from 44.6 ± 0.8 mN/m to 29.8 ± 1.8 mN/m. The results from the dilational interfacial rheology indicate that the effect of CYST is also greater in *P. aeruginosa* strains.

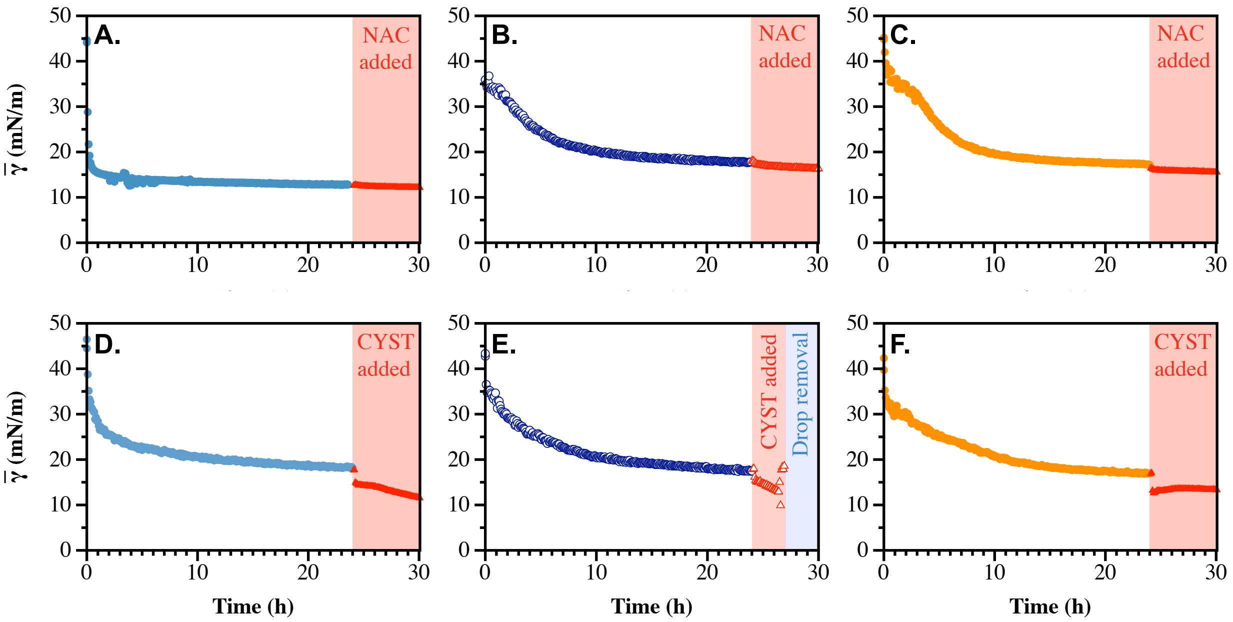

Figure S6. The effect of NAC and CYST on dynamic interfacial tension on monocultured films of wild type and mucoid *Pseudomonas aeruginosa* and *Staphylococcus aureus*. The IFT was recorded over a period of 24 h before either disrupting agent was added and then tracked over an additional 6 h after adding the drug. The effect of NAC was recorded on films of (A) PAO1, (B) PAO1*mucA22* and (C) SH1000. The effect of CYST was recorded on films of (D) PAO1, (E) PAO1*mucA22* and (C) SH1000.

References

[1] L.M. Filkins, J.A. Graber, D.G. Olson, E.L. Dolben, L.R. Lynd, S. Bhuju, G.A. O'Toole, Coculture of Staphylococcus aureus with Pseudomonas aeruginosa drives S. aureus towards fermentative metabolism and reduced viability in a cystic fibrosis model, Journal of bacteriology 197(14) (2015) 2252-2264.

[2] A. Hotterbeekx, S. Kumar-Singh, H. Goossens, S. Malhotra-Kumar, In vivo and In vitro Interactions between Pseudomonas aeruginosa and Staphylococcus spp, Frontiers in cellular and infection microbiology 7 (2017) 106.

[3] G.W. Lau, D.J. Hassett, H. Ran, F. Kong, The role of pyocyanin in Pseudomonas aeruginosa infection, Trends in molecular medicine 10(12) (2004) 599-606.
